# Moisture-induced crossover in the thermodynamic and mechanical response of hydrophilic biopolymer

**DOI:** 10.1007/s10570-019-02808-z

**Published:** 2019-10-31

**Authors:** Chi Zhang, Benoit Coasne, Robert Guyer, Dominique Derome, Jan Carmeliet

**Affiliations:** 1 Chair of Building Physics, Department of Mechanical and Process Engineering, ETH Zurich, 8093 Zurich, Switzerland; 2 Laboratory for Multiscale Studies in Building Physics, Swiss Federal Laboratories for Materials Science and Technology, Ueberlandstrasse 129, 8600 Duebendorf, Switzerland; 3 CNRS, LIPhy, Univ. Grenoble Alpes, 38000 Grenoble, France; 4 Department of Physics, University of Nevada, Reno, 1664 N. Virginia Street, Reno, NV 89557 USA

**Keywords:** Hemicellulose, Xylan, Molecular dynamics simulations, Moisture, Mechanics, Thermodynamics

## Abstract

The use of natural sustainable resources such as wood in green industrial processes is currently limited by our poor understanding of the impact of moisture on their thermodynamic and mechanical behaviors. Here, a molecular dynamics approach is used to investigate the physical response of a typical hydrophilic biopolymer in softwood hemicellulose—xylan—when subjected to moisture adsorption. A unique moisture-induced crossover is found in the thermodynamic and mechanical properties of this prototypical biopolymer with many quantities such as the heat of adsorption, heat capacity, thermal expansion and elastic moduli exhibiting a marked evolution change for a moisture content about 30 wt%. By investigating the microscopic structure of the confined water molecules and the polymer–water interfacial area, the molecular mechanism responsible for this crossover is shown to correspond to the formation of a double-layer adsorbed film along the amorphous polymeric chains. In addition to this moisture-induced crossover, many properties of the hydrated biopolymer are found to obey simple material models.

**Electronic supplementary material:**

The online version of this article (10.1007/s10570-019-02808-z) contains supplementary material, which is available to authorized users.

## Introduction

Wood and its various hydrophilic cellulosic components are the most abundant polymers on Earth (Arioli et al. 1998). While these compounds have been used through ages, a better understanding of the physical and chemical properties of such hydrophilic polymers would allow extending the range of applications of sustainable natural resources. As an important parameter to be taken into consideration, moisture strongly influences the mechanical and thermodynamic properties of hydrophilic biopolymers. In particular, it usually induces drastic changes of material dimension, stability and durability that can present both positive and negative effects. For instance, harnessed by quarry workers in ancient Egypt, the swelling pressure of wood due to moisture sorption was utilized to split stone (Bechthold and Weaver 2017). However, on the other hand, moisture uptake could lead to catastrophic biodegradation and failure of load-bearing wooden structures. From a fundamental viewpoint, important molecular mechanisms have been already unraveled such as those relevant to sorption hysteresis of deformable nanoporous materials (e.g. amorphous cellulose) (Chen et al. 2018). Yet, many important features regarding the role of moisture adsorption in the physical and mechanical responses of hydrophilic polymers remain to be identified to facilitate their implementation in fields such as food engineering (Li et al. 1998), biomedical device applications (Lyu and Untereker 2009) and architecture (Vailati et al. 2018).

Moisture changes the properties of polymer systems such as their stiffness (Harper and Rao 1994), glass transition (St. Lawrence et al. 2001) and crystallinity (Tanner et al. 1991). Numerous studies have reported strong evidence regarding the influence of moisture as a plasticization agent (Tanner et al. 1991; St. Lawrence et al. 2001; Perdomo et al. 2009; Carter and Schmidt 2012; Reuvers et al. 2013) that affects the dynamics of chains and the free volume in polymers (Gaylord and Van Wazer 1961). Most of these studies rely on macroscopic observations or limited microscopic information. To date, there is no general picture of polymer–moisture relationship at the microscopic (i.e. at the molecular) level. According to Refs. Hodge et al. (1996b), Li et al. (1998), Brouillet-Fourmann et al. (2002), water in materials can be in two possible states. At low moisture content, water competitively establishes hydrogen bonds (HBs) with polar hydroxyl groups of polymers and becomes the so-called “bound” or “unfreezable” water. At higher moisture content, “free water”, also referred to as “freezable water” or “freezable bound water”, is present, which further weakens the existing “bound” water–polymer interactions. This terminology has been used loosely. For example, some reports define “free” water in a view of phase change as the water which undergoes similar thermal phase transitions as bulk water (Hodge et al. 1996b). Some other reports define “free” water in terms of diffusion, as the water which is relatively free to travel through the microvoids and pores, and “bound” water as the water attached to the polar groups of the polymer (Alomayri et al. 2014). Some others define “free water” as the water in the wood cell lumen and “bound water” as the water held in cell wall material (Skaar 1988; Gezici-Koç et al. 2017). Part of the reason for this ambiguity is the limited resolution of current experimental techniques. It is important however to access the microscopic information, e.g. differentiating the states of water in the material, for heterogeneous hydrated polymer systems, which presently still remains an open question.

In this study, we follow up on the idea of different states of water but do not follow the ambiguous terminology. Here, we use molecular dynamics (MD) to investigate the role of molecular interactions, e.g. HBs, which are critical to explaining the microscopic mechanisms leading to the coupling between the polymer properties and water adsorption. MD has shown its capability of simulating cellulosic material, such as cellulose and xylan, in agreement with existing experimental studies (Zhao et al. 2014; Kulasinski et al. 2016). Here the arabinoglucuronoxylan (AGX), one of the most abundant hemicelluloses of softwood (Reid 1997), is chosen as a prototypical model of hydrophilic biopolymers in wood. This specific yet representative compound is involved in numerous applications, such as packaging (Escalante et al. 2012), biomedical products (Li et al. 2011), plastic additives (Ünlü et al. 2009), and etc., as summarized by Sedlmeyer (2011). Through the investigation of various mechanical and thermodynamic properties of AGX, we show that all material properties undergo a clear transition (later in the microscopic sorption analysis referred to as a crossover) in moisture sensitivity at the same transition (crossover) point of ~ 30 wt% moisture content. Analysis of water microscopic structure shows that, when the first adsorbed layer saturates, bulk-like water clusters start to grow at the transition point. This differentiation between water adsorbed in the first layer and beyond enables us to establish material models which show that the mechanical and thermodynamic properties of the hydrated biopolymer depend in a simple fashion on the different adsorption populations.

## Materials and methods

### Modeling of xylan

**Fig. 1:**
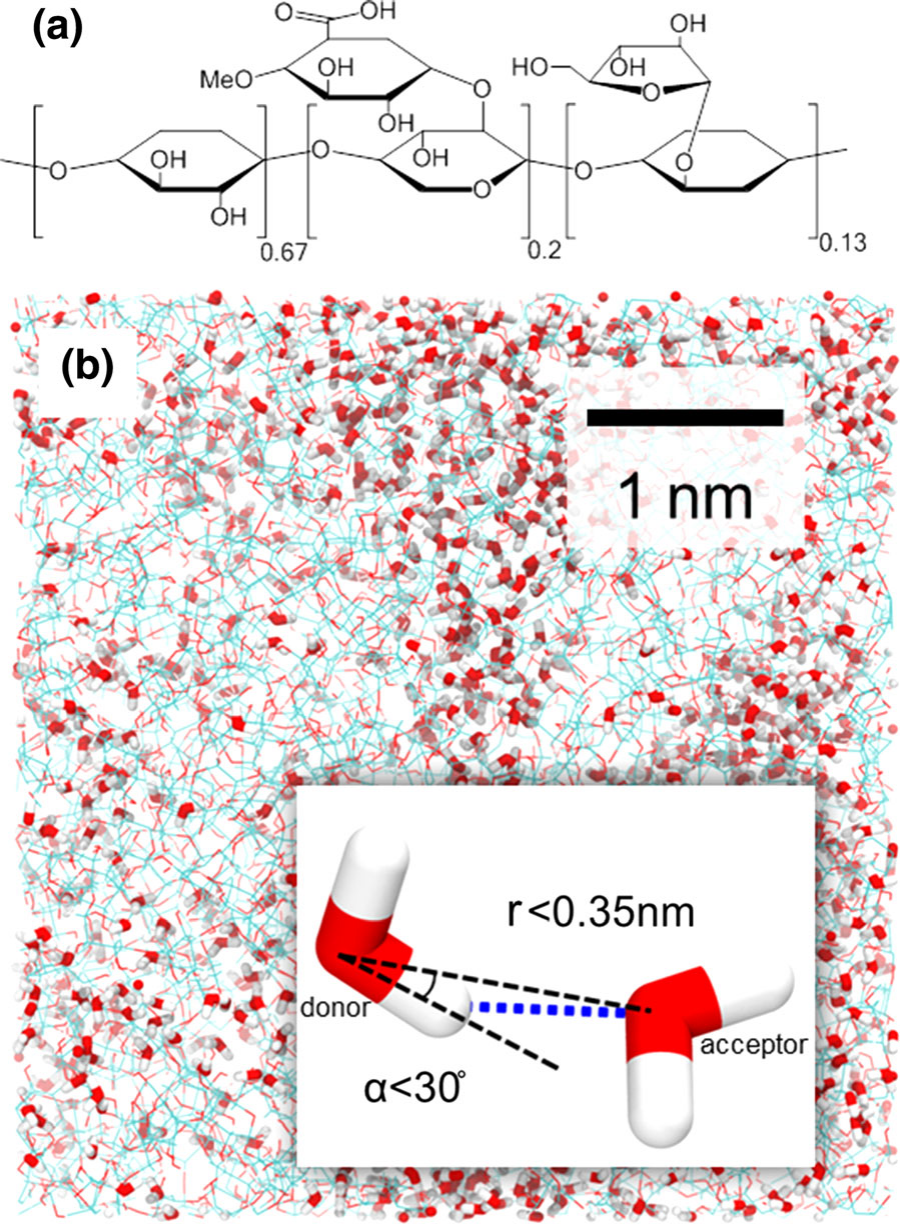
**a** Chemical structure of AGX consisting of 67% xylose, 20% glucuronoacid-xylose and 13% arabinoxylose. **b** Typical molecular configuration of hydrated polymer system at a moisture content *m* = 18% with carbon atoms in cyan, oxygen atoms in red and hydrogen atoms in white. The inset illustrates the criteria to define hydrogen bonds, $r < 0.35\;{\text{nm}}$ and $\alpha < 30^{ \circ }$

AGX is formed by a backbone of β-1,4-linked β-D-xylopyranose units, partially substituted at O-2 by 4-O-methyl-α-d-glucopyranosyluronic acid and at O-3 by α-l-arabinofuranose (Reid 1997) [with the degree of substitution depending on botanic sources and extraction methods (Den Haan and Van Zyl 2003)]. In the present work, a polymer made up of 67% xylose, 20% glucuronoacid-xylose and 13% arabinoxylose is used (chemical structure shown in Fig. 1a).

In MD, molecular interactions are numerically modeled by a force field, i.e. mathematical functions fitted from experimental measurements with necessary simplifications and assumptions. In this study, the force field parameters, as well as the geometry of AGX monomers, are obtained from the automated topology builder (Malde et al. 2011) with Gromos 53a6 force field (Oostenbrink et al. 2004). Multistep quantum mechanics calculations are carried out to optimize the geometry of the monomers and harvesting the force filed parameters such as equilibrium bond length, angles and etc. For the detailed workflow of the automated topology builder, we refer to Ref. Malde et al. (2011). The three types of monomers are constructed using Material Studio 8.0 and randomly polymerized into chains with a degree of polymerization of 100 (Gorshkova et al. 2012). Gromacs 2016 software (Berendsen et al. 1995; Abraham et al. 2015) is used for simulation. Five chains of AGX are inserted randomly into a periodic box, with periodic boundary conditions in the three principal directions to avoid finite-size effects. The dry system finally reaches a size of about 5 × 5 × 5 nm^3^ and a density of 1.3 g/cm^3^. The density is in accordance with the literature, as measured for a xylan powder extracted from corn cobs (Verbeek 2012). Following the same procedure, two additional systems are prepared and then investigated to improve the statistics and obtain data more representative of disorder in real systems. The three replicas differ by their orientation and arrangement of chains. A more detailed description of modeling methods, validation and investigation are included in the supporting information.

### Adsorption process and measurements of material properties

Starting with the dry system, single point charge (SPC) (Berendsen et al. 1987) water molecules are inserted randomly one after another into the simulation box. Special attention is paid to avoid overlap with the polymer and previously inserted water molecules. Each insertion is followed by energy minimization and a relaxation run of 100 ps. Besides the aforementioned density, the atomistic models are subject to validation through the comparison with available experimental results, i.e. sorption isotherm, isotropy and swelling strain, where good agreement was found, justifying the validity of the preparation of initial structure and adsorption process. Due to computational costs, we choose to report results at 20 moisture content levels. Figure 1b shows a typical molecular configuration of the hydrated system at *m* = 18%, where carbon atoms in cyan, oxygen atoms in red and hydrogen atoms in white. Polymer and water molecules are shown in thin lines and thick sticks, respectively. The moisture content in this study is defined as the mass of water divided by the mass of dry material: *m* = *m*_w_/*m*_p_. Integral heat of adsorption, thermal expansion coefficient, heat capacity, elastic constants and Poisson’s ratio are then measured as a function of moisture content using the method documented in the supporting information. Similar to experimental studies where each physical property is derived from an independent measurement, most of the properties in this study are measured with separate simulations, as they require different loading conditions, resulting in separate trajectories for post-processing. Thermal expansion coefficient and heat capacity are measured with the same trajectory, however, three repetitions are employed. As will be shown below, all these properties show a transition happening at *m* ~ 30% which, to the best of our knowledge, has not yet been reported.

## Results and discussions

### Properties as a function of moisture content and occurrence of moisture-induced crossover

**Fig. 2:**
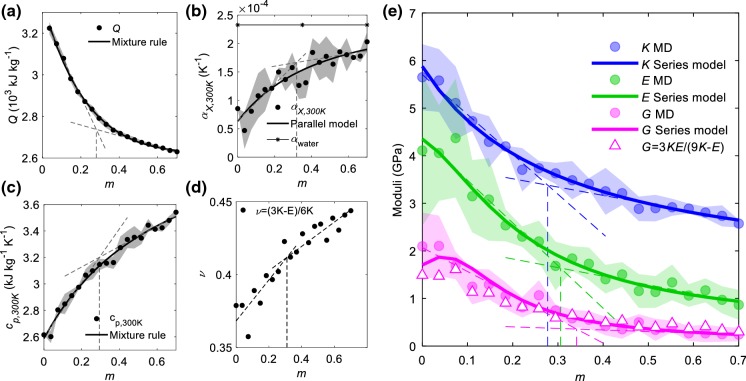
Material properties of AGX as a function of moisture content at room temperature 300 K. The vertical dashed lines denote the transition at around *m* ~ 30%. **a** Heat of adsorption *Q*(*m*). **b** Uniaxial thermal expansion coefficient $\alpha_{{X,300\,{\text{K}}}} \;\left( {\text{m}} \right)$. **c** Heat capacity $C_{{p,300\;{\text{K}}}} \;\left( {\text{m}} \right)$. **d** Poisson’s ratio with the transition at around *m* ~ 30%. **e** Elastic constants, i.e. bulk, Young’s and shear moduli

The integral heat of adsorption *Q*(*m*) is shown in Fig. 2a. The dots are the arithmetical average of the three different models while the shaded areas denote the standard deviation (similar representation is used for Fig. 2b, c, e). At low moisture content, the heat of adsorption is close to that measured using MD for amorphous cellulose (AC), ~ 3620 kJ/kg (Kulasinski 2015). At high moisture content, the heat of adsorption gradually approaches the value of bulk water, ~ 2260 kJ/kg (Murphy and Koop 2005). Interestingly, two different linear regimes can be identified, one for low moisture content and another for high moisture content. The transition separating the two regimes occurs around *m* ~ 30%.

The uniaxial thermal expansion coefficient of AGX is shown in Fig. 2b. As moisture increases, the thermal expansion coefficient of AGX approaches the value of pure SPC water indicated by the solid line with asterisks. At 300 K, the uniaxial thermal expansion coefficient of SPC water is $\alpha_{{X,300\;{\text{K}},SPC}} = 2.3 \times 10^{ - 4} \;{\text{K}}^{ - 1} ,$ which is larger than the experimental value ~ 7.9 × 10^−5^ K^−1^ (a known imperfection of the SPC water model (Jorgensen and Jenson 1998)). Like for the heat of adsorption, a transition occurs around 30% moisture content. The heat capacity of AGX is shown in Fig. 2c. With increasing moisture content, the heat capacity approaches the value of pure SPC water. At 300 K, the heat capacity of SPC water is $C_{{p,300\;{\text{K}}}} = 4.594\;{\text{kJ/}}({\text{kg}}\;{\text{K}}),$ which agrees well with the experimental value of 4.186 kJ/(kg K) (Chase Jr. 1998). Once more, we note a transition occurring around 30% moisture content. The bulk, Young’s and shear moduli and Poisson’s ratio of AGX are shown as solid dots in Fig. 2d, e. The bulk and shear moduli are difficult to measure experimentally, as samples are usually prepared in the form of thin films (Chang et al. 2018). Available reports, mostly about Young’s moduli at 27 °C and 50% RH, agree well with our simulation which is about 2.5 GPa under similar temperature and moisture level (Gröndahl et al. 2004; Höije et al. 2005; Escalante et al. 2012). For all these mechanical properties, there is also a transition happening around *m* ~ 30%.

For a homogeneous isotropic material, like AGX, shear moduli can be predicted from bulk and Young’s moduli using (1). It is important to note that the dry material is found to be isotropic (see the supporting information), while the hydrated material may show some deviations of isotropy. However, we can assume that the materials remain mainly isotropic and (1) is assumed to be valid. The predicted values represented by white triangles in Fig. 2e agree well with the shear moduli measured by MD (pink dots in Fig. 2e).

1 $$G = \frac{3KE}{9K - E}$$

### Mechanisms of moisture-induced crossover

#### Density of polymer–polymer and polymer–water hydrogen bonds

**Fig. 3:**
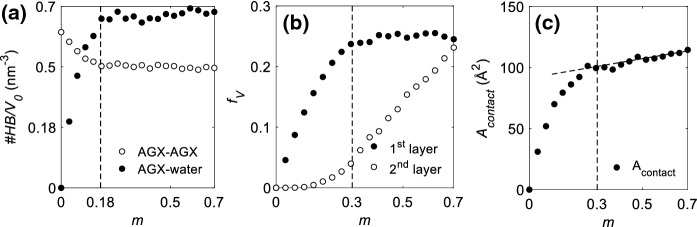
**a** Density of hydrogen bonds between AGX–AGX (white) and AGX–water (black). **b** Volume fraction of 1st (black) and 2nd (white) layers of water. At *m* = 0.3, the 1st layer saturates while the 2nd layer starts to quickly grow. **c** Contact area between polymer and water

To explain the transition behavior observed around *m* ~ 30%, the hydrogen bond network and water distribution in the hydrated AGX are now discussed. The HBs between polymer chains play a central role in the moisture-induced effects of hydrophilic polymeric material, such as weakening (Kulasinski et al. 2015) and hysteresis (Chen et al. 2018). The establishment of HBs is judged by applying the criteria referred to in Refs. Soper and Phillips (1986), Teixeira and Bellissent-Funel (1990), Luzar and Chandler (1993) and shown in the illustration in the inset of Fig. 1b, i.e. $r < 0.35\;{\text{nm}}$ and $\alpha < 30^{ \circ } ,$ where *r* is the distance between the donor oxygen atom and the acceptor oxygen atom and $\alpha$ is the angle of acceptor oxygen atom—donor oxygen atom—donor hydrogen atom. The number of HBs is normalized by the initial dry material volume *V*_0_ to obtain the density of polymer–polymer HBs (*#HB/V*_0_). Both *#HB/V*_0_ for AGX–AGX and AGX–water—shown in black and white dots respectively in Fig. 3a—remain constant after *m* > 18%, which is below the transition point of *m* ~ 30%. The number of HBs cannot explain the crossover observed for the material properties in Fig. 2.

In previous work, for another wood polymer, amorphous cellulose (AC), a linear relationship between the number of polymer–polymer HBs and the mechanical moduli was found. The breakage of HBs was then understood to be the main mechanism of the mechanical weakening of AC (Kulasinski et al. 2015). In the present study, within the range of 0 ~ 18% moisture content, moduli also scale linearly with the HBs breakage. However, for higher moisture contents, i.e. *m* > 18%, further decrease of moduli is seen to occur without further breaking of HBs. This indicates that there must be another mechanism explaining the mechanical weakening behavior of AGX for *m* > 18%, and the crossover happening around *m* ~ 30%. It is abnormal to find that hydrogen bond is not dominating such important behaviors of the hydrophilic polymer.

#### Double layer adsorption

**Fig. 4:**
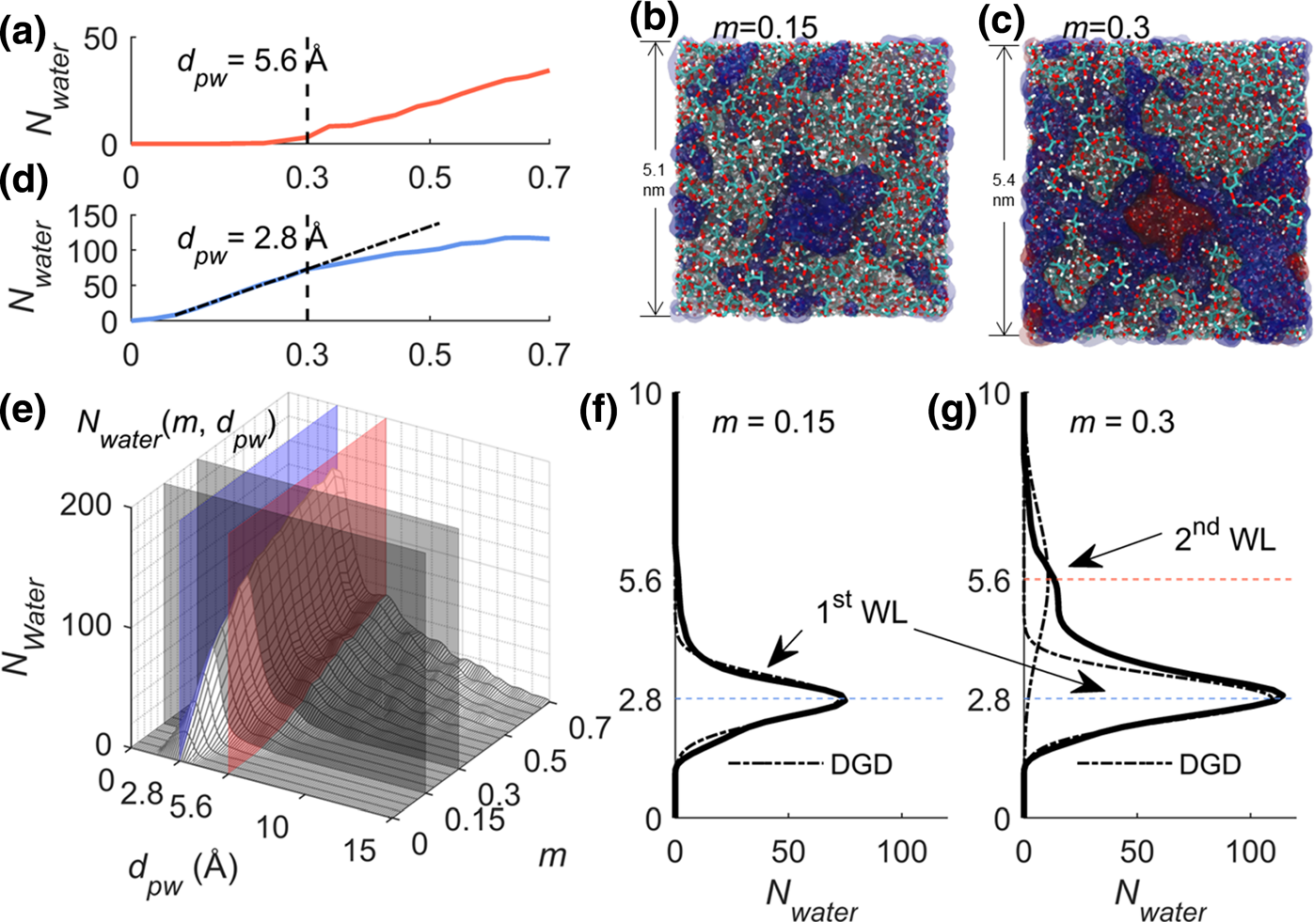
**a** Water population as a function of moisture content *N*_*water*_(*m*) at the polymer–water distance of 5.6 Å. **b**, **c** Juxtaposing snapshots of hydrated polymer system at *m* = 0.15 and 0.3, respectively. The polymer chains, the 1st and 2nd layer of water are shown in color thick sticks, blue and red surfaces, respectively. **d** Water population *N*_*water*_(*m*) as a function of moisture content *m* at *d*_*pw*_ = 2.8 Å. **e** Water population as a function of moisture content and polymer–water distance *N*_*water*_(*m*, *d*_*pw*_) shown as a 3D surface. The four cut planes, i.e. *d*_*pw*_ = 5.6 Å and 2.8 Å, *m* = 0.15 and 0.3, refer to the coordinate planes of subplot **a**, **d**, **f** and **g** respectively. **f** and **g** Water population as a function of polymer–water distance at *m* = 0.15 and 0.3, respectively. The black dashed curves are double Gaussian decomposition (DGD) of water population

We explore another possible mechanism, inspired by the observation that further moisture-induced mechanical weakening occurs at the appearance of “free water”. As formerly mentioned, water in the material can be in two possible states (Hodge et al. 1996b; Li et al. 1998; Brouillet-Fourmann et al. 2002), i.e. “bound” or “unfreezable” and “free” or “freezable”. Thanks to MD, the trajectories of individual water molecules can be tracked. We propose a statistical description of adsorption layers, where the distance between a water molecule and the nearest polymer atom (*d*_*pw*_) is used. The water population along the polymer chains is given as a function of the polymer–water distance and moisture content (the surface plot in Fig. 4e). The polymer–water distance *d*_*PW*_ is defined as the distance between the oxygen atom of water and its nearest polymer neighbor atom. The water population *N*_*Water*_(*m,d*_*pw*_) is the time average of the number of water molecules at a specific *m* and *d*_*PW*_. From Fig. 4e, we observe that, at lower moisture content, there is only one peak centered at 2.8 Å. At higher moisture content and practically for *m* > 30%, at least two major peaks, centered at 2.8 Å and 5.6 Å, can be identified. Between the two peaks, there is a “valley”, i.e. a local minimum, at 4.5 Å. Water population is practically zero for *d*_*PW*_ > 15 Å. We define the water that resides within 4.5 Å of the polymer chains as the 1st adsorbed water layer and water from 4.5 to 20 Å as the 2nd adsorbed water layer. The occurrence of double-layer adsorption is also supported by the sigmoidal shape of the AGX sorption isotherm (type II isotherm) (Ergun et al. 2010). The double-layer adsorption starts from around *m* ~ 30%.

The blue and red cut planes in Fig. 4e denote the center location of the 1st and 2nd adsorbed water layers, respectively. The solid curves in Fig. 4a, d are the water population at *d*_*pw*_ = 5.6 Å and 2.8 Å, which refers to the intersection between the cut planes and the surface in Fig. 4e. As shown in Fig. 4a, the water population starts to grow from *m* = 0.3. As shown in Fig. 4d, the linear growth of water population slows down from *m* = 0.3, indicated by the divergence of the solid blue curve below the dashed line fitted from the range of low moisture content. The surface in Fig. 4e can also be cut by the planes of *m* = 0.15 and 0.3, for which we get the solid water population curves in Fig. 4f, g. For *m* = 0.15, as shown in Fig. 4f, the water population only has one single peak around 2.8 Å. However, for *m* = 0.3, shown in Fig. 4g, two peaks (2.8 Å and 5.6 Å) can be identified. The water population can be described by the summation of two Gaussian distributions, as shown by the dashed lines calculated from double Gaussian decomposition (DGD). Figure 4b, c are juxtaposing snapshots of the hydrated polymer system at *m* = 0.15 and 0.3, respectively. The polymer chains, the 1st and the 2nd layer of water are shown in color thick sticks, blue and red surfaces, respectively. The average side length of the system increases from 5.1 nm for *m* = 0.15 to 5.4 nm for *m* = 0.3 indicating swelling of the system.

Figure 3b shows the volume fraction of the 1st and 2nd water layers as a function of moisture content. At *m* ~ 30%, the 1st layer saturates. The 2nd water layer starts to grow quickly from *m* ~ 30%, though it already started to emerge before this moisture level. The saturation of the 1st water layer is supported by the analysis of the contact area between polymer and water shown in Fig. 3c. The water–polymer contact area is defined as $$A_{contact} = A^{polymer} + A^{water} - A^{system} ,$$ where $$A^{polymer} ,$$$$A^{water}$$ and $$A^{system}$$ are the surface areas of the polymer, water and full system. These surface areas are measured by the so-called rolling ball algorithm (Shrake and Rupley 1973), using a ball, of a specific radius (here 1 Å), to roll along the surfaces of interest, namely the surfaces made by the van der Waals surface of the atoms. The area of the plane defined by the center of the ball as it rolls is the calculated surface area. As shown in Fig. 3c, within the moisture range of 0–30%, water adsorption generates new contact surface. This means that the newly adsorbed water molecules bind to the polymer. However, for *m* > 30%, the contact area saturates, which means that the newly adsorbed water molecules attach themselves to the formerly adsorbed water rather than to the polymer.

As the 1st and 2nd water layers are located at a different distance from the polymer chain, their interactions with the polymer are also different. This induces different dynamics and mobility for these two populations (Li et al. 1998; Zhao et al. 2019). The crossover from one to two layers of water is likely to influence the properties of the hydrated polymer and to induce a transition, i.e. crossover, in thermodynamic and mechanical properties of the polymer. This new insight provides a long-time missing piece of information for the full understanding of the structure–property relationship of polymers, which may interest many research fields and industries. For example, in the plastic, adhesive, hydrogel and food industries, the states and molecular structures of the water within polymers matrix have an important impact (Hodge et al. 1996a; Li et al. 1998; Brouillet-Fourmann et al. 2002) on the material properties where crossover might come into play. Another example is that, in classic linear poromechanics (Coussy 2003), the coupling energy term is written as the linear summation of the external variables, meaning that the poroelastic properties, e.g. bulk modulus, are constants, which might lead to wrong predictions. This study shows that two modes, separated by the crossover point, exist in the moisture–material interactions. This physics could provide guidance on the proper modeling of thermodynamic and mechanical properties of polymeric porous media thus facilitating the improvement of existing poromechanical models.

Interestingly, in experiments, similar saturations around *m* ~ 30% have been also found for other materials. Li et al. (1998) were able to differentiate “unfreezable” water, which creates strong interaction with polymer either energetically bounded or kinetically retarded, to “freezable” water by using differential scanning calorimetry (DSC). They found that the saturation of “unfreezable” water happens at 30% moisture content. Brouillet-Fourmann et al. (2002) revealed a similar saturation of bound water at 30% moisture content in a hydrated starch system also by DSC. Hodge et al. (1996a) found a saturation of “nonfreezing water” for water concentration greater than 30% in polyvinyl alcohol.

#### Properties of polymer, first and second layers of water predicted by material models

As shown in Fig. 2a, the adsorption of the first water molecules releases more heat than adsorption of those of the 2nd layer. After *m* > 30%, the 2nd layer of water quickly grows and starts to dominate the heat of adsorption. The heat of adsorption approaches the latent heat of liquid water as moisture increases, indicating that the fast-growing 2nd water layer resembles bulk water. Similarly in the case of heat capacity, thermal expansion coefficient, elastic constants and Poisson’s ratio, the saturation of the 1st layer of water and the quick growth of the 2nd layer of water induces the crossover. The differentiation of two layers of water enables us to decompose properties into the contributions of different types of water. Here we adopt three simple material models, i.e. mixture rule, parallel and series models, to predict the mechanical and thermodynamic properties measured, which are seen as the summation of the contributions of three major types of materials involved: polymer, 1st and 2nd layers of water. The constitutive equations for these models read:

2 $${\text{Mixture}}\;{\text{rule/Parallel}}\quad X_{c} = f_{v,p} X_{p} + f_{v,w1} X_{w1} + f_{v,w2} X_{w2}$$

3 $${\text{Series}}\quad 1 /X_{c} = f_{v,p} /X_{p} + f_{v,w1} /X_{w1} + f_{v,w2} /X_{w2}$$

where $X_{c}$, $X_{p}$, $X_{w1}$, $X_{w2}$, $f_{v,p}$, $f_{v,w1}$ and $f_{v,w2}$ are the properties of composite, polymer, 1st and 2nd layer of water, the volume fraction of polymer, 1st and 2nd layer of water respectively. From simulations, $X_{c}$, $f_{v,p}$, $f_{v,w1}$ and $f_{v,w2}$ can be directly extracted, however $X_{p}$, $X_{w1}$ and $X_{w2}$ are parameters to be determined through fitting analysis. The mixture rule and the parallel model share the same mathematical equations, but they differ in their interpretation. The mixture rule represents the composite behavior of scalar properties such as heat of adsorption and heat capacity, while mechanical properties, such as Young’s, bulk, shear moduli and thermal expansion coefficient are tensorial properties represented by a parallel or series models.

The mechanical moduli, i.e. bulk, Young’s and shear moduli, are found to correspond to a series model, the heat of adsorption and heat capacity correspond to the mixture rule, and the thermal expansion coefficient corresponds to a parallel model. Figure 2 gives the data obtained by MD and the fitted models (solid curves). Moreover, it is found that $X_{p}$ and $X_{w2}$ correspond relatively well with the value of dry polymer and bulk water. Under the same context, it is reasonable to speculate that $X_{w1}$ represents the properties of the 1st layer of water, which are difficult to measure both numerically and experimentally. A list of values for $X_{p}$, $X_{w1}$ and $X_{w2}$ are summarized in Table 1.

**Table 1:** Predicted properties of the polymer, first and second layers of water

	Polymer	First layer of water	Second layer of water	Bulk water	Unit
Bulk moduli (*K*)	5.9	1.9	1.5	1.7	GPa
Young’s moduli (*E*)	4.6	1.1	0.32	0	GPa
Shear moduli (*G*)	1.9	0.6	0.068	0	GPa
Adsorption heat (*Q*)	3340	1110	2660	2260	kJ/kg
Thermal expansion coefficient $(\alpha )$	6.4 × 10^−5^	3.7 × 10^−4^	2.9 × 10^−4^	2.33 × 10^−4^	1/K
Heat capacity $(C_{p} )$	2.6	4.6	4.4	4.594	kJ/kg/K

Bulk, Young’s and shear moduli are inferred by the series model. Adsorption heat and heat capacity are inferred by the mixture rule. Thermal expansion coefficient is inferred by the parallel model. The properties of bulk water are also included to be compared with those of the second layer of water

Finding that material properties like the heat of adsorption and the heat capacity follow a mixture rule model is logical, since these are scalar properties. Our findings show that the polymer material, first and second adsorbed water layer act as a layered composite material loaded mainly normally to the layers as in a series model. The parallel model shows that the polymers, as well as layers of water, mainly expand along their longest direction which is along the layer direction as in a parallel model. According to all these analyses, we conclude that the saturation of the 1st water layer and the development of the 2nd water layer is the mechanism inducing the crossover occurring around *m* ~ 30% (as seen in heat of adsorption, thermal expansion coefficient, heat capacity, elastic moduli and Poisson’s ratio).

## Conclusions

There is considerable interest in understanding the thermodynamic and mechanical responses of hydrophilic plant biopolymers upon moisture adsorption. In particular, this could allow better utilizing plant-based sustainable resources. In this study, molecular dynamics simulation is used to investigate the influence of moisture on the properties of a prototypical hydrophilic polymer: softwood xylan. The heat of adsorption, heat capacity, thermal expansion, elastic moduli and Poisson’s ratio are yielded as a function of moisture content. All these mechanical and thermodynamic properties show a crossover occurring around 30 wt% moisture content, which cannot be explained by the alteration of the hydrogen bond network which plateaus at 18 wt%. A study of water population distribution and polymer–water contact area leads to the identification of a double-layer adsorption. The saturation of the first adsorption layer and the growth of the second adsorption layer of water are identified as the main mechanism of the moisture-induced crossover. This decomposition of two types of water also enables a further material model study, where properties of the composite can be attributed to the contributions of the three major components, i.e. polymer, first and second layer of water. The universality and diversity of the observed crossover in the current study still require further investigation. Nevertheless, the simulation framework and the statistical analysis of the layering structure of water could be extended to other material systems potentially explaining phenomenon associated with it. These new insights provide an important missing piece of information for the full understanding of the structure–property relationship of polymers, which may interest many research fields and industries.

## Supporting information

The detailed description of modeling and measurement methods.

## Electronic supplementary material

Below is the link to the electronic supplementary material.

Supplementary material 1 (DOCX 87 kb)

## References

[CR1] Abraham MJ, Murtola T, Schulz R (2015). Gromacs: high performance molecular simulations through multi-level parallelism from laptops to supercomputers. SoftwareX.

[CR2] Alomayri T, Assaedi H, Shaikh FUA, Low IM (2014). Effect of water absorption on the mechanical properties of cotton fabric-reinforced geopolymer composites. J Asian Ceram Soc.

[CR3] Arioli T, Peng L, Betzner AS (1998). Molecular analysis of cellulose biosynthesis in arabidopsis. Science (80-).

[CR4] Bechthold M, Weaver JC (2017). Materials science and architecture. Nat Rev Mater.

[CR5] Berendsen HJC, Grigera JR, Straatsma TP (1987). The missing term in effective pair potentials. J Phys Chem.

[CR6] Berendsen HJC, van der Spoel D, van Drunen R (1995). GROMACS: a message-passing parallel molecular dynamics implementation. Comput Phys Commun.

[CR7] Brouillet-Fourmann S, Carrot C, Lacabanne C (2002). Evolution of interactions between water and native corn starch as a function of moisture content. J Appl Polym Sci.

[CR8] Carter BP, Schmidt SJ (2012). Developments in glass transition determination in foods using moisture sorption isotherms. Food Chem.

[CR9] Chang J, Toga KB, Paulsen JD (2018). Thickness dependence of the Young’s modulus of polymer thin films. Macromolecules.

[CR10] Chase Jr. MW (1998) Nist-JANAF thermochemical tables, 4th edn. https://www.nist.gov/publications/nist-janaf-thermochemical-tables-4th-edition

[CR11] Chen M, Coasne B, Guyer R (2018). Role of hydrogen bonding in hysteresis observed in sorption-induced swelling of soft nanoporous polymers. Nat Commun.

[CR12] Coussy O (2003). Poromechanics.

[CR13] Den Haan R, Van Zyl WH (2003). Enhanced xylan degradation and utilisation by *Pichia stipitis* overproducing fungal xylanolytic enzymes. Enzyme Microb Technol.

[CR14] Ergun R, Lietha R, Hartel RW (2010). Moisture and shelf life in sugar confections. Crit Rev Food Sci Nutr.

[CR15] Escalante A, Gonçalves A, Bodin A (2012). Flexible oxygen barrier films from spruce xylan. Carbohydr Polym.

[CR16] Gaylord NG, Van Wazer JR (1961) Viscoelastic properties of polymers. John D. Ferry. Wiley, New York, xx + 482 pp $15.00

[CR17] Gezici-Koç Ö, Erich SJF, Huinink HP (2017). Bound and free water distribution in wood during water uptake and drying as measured by 1D magnetic resonance imaging. Cellulose.

[CR18] Gorshkova T, Brutch N, Chabbert B (2012). Plant fiber formation: state of the art, recent and expected progress, and open questions. CRC Crit Rev Plant Sci.

[CR19] Gröndahl M, Eriksson L, Gatenholm P (2004). Material properties of plasticized hardwood xylans for potential application as oxygen barrier films. Biomacromolecules.

[CR20] Harper BD, Rao JM (1994). Some effects of water immersion on the mechanical behavior of a polyimide film. J Electron Packag.

[CR21] Hodge RM, Bastow TJ, Edward GH (1996). Free volume and the mechanism of plasticization in water-swollen poly(vinyl alcohol). Macromolecules.

[CR22] Hodge RM, Edward GH, Simon GP (1996). Water absorption and states of water in semicrystalline poly(vinyl alcohol) films. Polymer.

[CR23] Höije A, Gröndahl M, Tømmeraas K, Gatenholm P (2005). Isolation and characterization of physicochemical and material properties of arabinoxylans from barley husks. Carbohydr Polym.

[CR24] Jorgensen WL, Jenson C (1998). Temperature dependence of TIP3P, SPC, and TIP4P water from NPT Monte Carlo simulations: seeking temperatures of maximum density. J Comput Chem.

[CR25] Kulasinski K (2015) Physical and mechanical aspects of moisture adsorption in wood biopolymers investigated with atomistic simulations. 10.3929/ethz-a-010564673

[CR26] Kulasinski K, Guyer R, Keten S (2015). Impact of moisture adsorption on structure and physical properties of amorphous biopolymers. Macromolecules.

[CR27] Kulasinski K, Salmén L, Derome D, Carmeliet J (2016). Moisture adsorption of glucomannan and xylan hemicelluloses. Cellulose.

[CR28] Li S, Dickinson LC, Chinachoti P (1998). Mobility of “unfreezable” and “freezable” water in waxy corn starch by 2 H and 1 H NMR. J Agric Food Chem.

[CR29] Li X, Shi X, Wang M, Du Y (2011). Xylan chitosan conjugate—a potential food preservative. Food Chem.

[CR30] Luzar A, Chandler D (1993). Structure and hydrogen bond dynamics of water–dimethyl sulfoxide mixtures by computer simulations. J Chem Phys.

[CR31] Lyu S, Untereker D (2009). Degradability of polymers for implantable biomedical devices. Int J Mol Sci.

[CR32] Malde AK, Zuo L, Breeze M (2011). An automated force field topology builder (ATB) and repository: version 1.0. J Chem Theory Comput.

[CR33] Murphy DM, Koop T (2005). Review of the vapour pressures of ice and supercooled water for atmospheric applications. Q J R Meteorol Soc.

[CR34] Oostenbrink C, Villa A, Mark AE, Van Gunsteren WF (2004). A biomolecular force field based on the free enthalpy of hydration and solvation: the GROMOS force-field parameter sets 53A5 and 53A6. J Comput Chem.

[CR35] Perdomo J, Cova A, Sandoval AJ (2009). Glass transition temperatures and water sorption isotherms of cassava starch. Carbohydr Polym.

[CR36] Reid JSG (1997) Carbohydrate metabolism: structural carbohydrates. In: Plant biochemistry. Elsevier, pp 205–236

[CR37] Reuvers N, Huinink H, Adan O (2013). Water Plasticizes only a small part of the amorphous phase in nylon-6. Macromol Rapid Commun.

[CR38] Sedlmeyer FB (2011). Xylan as by-product of biorefineries: characteristics and potential use for food applications. Food Hydrocolloids.

[CR39] Shrake A, Rupley JA (1973). Environment and exposure to solvent of protein atoms. Lysozyme and insulin. J Mol Biol.

[CR40] Skaar C (1988). Wood–water relations.

[CR41] Soper AK, Phillips MG (1986). A new determination of the structure of water at 25°C. Chem Phys.

[CR42] St. Lawrence S, Willett JL, Carriere CJ (2001). Effect of moisture on the tensile properties of poly(hydroxy ester ether). Polymer.

[CR43] Tanner SF, Hills BP, Parker R (1991). Interactions of sorbed water with starch studied using proton nuclear magnetic resonance spectroscopy. J Chem Soc Faraday Trans.

[CR44] Teixeira J, Bellissent-Funel MC (1990). Dynamics of water studied by neutron scattering. J Phys Condens Matter.

[CR45] Ünlü CH, Günister E, Atici O (2009). Synthesis and characterization of NaMt biocomposites with corn cob xylan in aqueous media. Carbohydr Polym.

[CR46] Vailati C, Bachtiar E, Hass P (2018). An autonomous shading system based on coupled wood bilayer elements. Energy Build.

[CR47] Verbeek C (2012). Products and applications of biopolymers.

[CR48] Zhao Z, Crespi VH, Kubicki JD (2014). Molecular dynamics simulation study of xyloglucan adsorption on cellulose surfaces: effects of surface hydrophobicity and side-chain variation. Cellulose.

[CR49] Zhao H, Chen Z, Du X, Chen L (2019). Contribution of different state of adsorbed water to the sub-Tg dynamics of cellulose. Carbohydr Polym.

