# Supplementary material for: Moisture-induced crossover in the thermodynamic and mechanical response of hydrophilic biopolymer
Source: Cellulose (Lond). 2019 Oct 31;27(1):89–99. doi: 10.1007/s10570-019-02808-z (PMC6960215; doi:10.1007/s10570-019-02808-z)
Supplement: Supplementary file 1 — Supplementary material 1 (DOCX 87 kb) [file 10570_2019_2808_MOESM1_ESM.docx]

**Supporting Information**

Moisture-Induced Crossover in the Thermodynamic and Mechanical Response of Hydrophilic Biopolymer

*Chi Zhang^†^*[*^§^*](https://pubs.acs.org/doi/full/10.1021/acs.jpclett.9b00416#notes2)*, Benoit Coasne^‡*^, Robert Guyer^#^, Dominique Derome*[*^§^*](https://pubs.acs.org/doi/full/10.1021/acs.jpclett.9b00416#notes2)*, Jan Carmeliet^†*^*

^†^ Chair of Building Physics, Department of Mechanical and Process Engineering, ETH Zurich, 8093, Zurich, Switzerland

[^§^](https://pubs.acs.org/doi/full/10.1021/acs.jpclett.9b00416#notes2) Laboratory for Multiscale Studies in Building Physics, Swiss Federal Laboratories for Materials Science and Technology, Ueberlandstrasse 129, 8600, Duebendorf, Switzerland

^‡^ Univ. Grenoble Alpes, CNRS, LIPhy, 38000 Grenoble, France

^#^ Department of Physics, University of Nevada, Reno, 1664 N. Virginia Street, Reno, NV, 89557, USA

^*^Corresponding author's e-mail address: [benoit.coasne@univ-grenoble-alpes.fr](mailto:benoit.coasne@univ-grenoble-alpes.fr); [cajan@ethz.ch](mailto:cajan@ethz.ch)

Modeling methods

**Chemical structure and atomistic model.** Arabinoglucuronoxylan (AGX), the most common type of xylan in softwood(Reid 1997), is formed by a backbone of β-1,4-linked β-D-xylopyranose units, partially substituted at O-2 by 4-O-methyl-α-D-glucopyranosyluronic acid and at O-3 by α-L-arabinofuranose(Reid 1997) (with the degree of substitution depending on botanic sources and extraction methods(Den Haan and Van Zyl 2003)). Generally, AGX from endospermic material is highly substituted, while AGX from hardwood and in the lignified tissues of grasses and cereals has less arabinose(Luonteri et al. 1995). Enzymatically derived AGX from sugi, a softwood, shows the presence of regions of eight contiguous unsubstituted D-xylopyranose units(Yamasaki et al. 2011). Matrix-assisted laser desorption/ionization mass spectrometry analysis of oligosaccharide mixtures of AGX from spruce, pine and larch, shows that, for most softwood AGX, 4-O-methyl-glucuronic acid residues are distributed every seventh or eighth xylopyranose residue(Jacobs et al. 2001). Conifer xylan is found to be decorated with methyl-glucuronic acid residues on every sixth xylosyl residue, while arabinose and methyl-glucuronic acid residues are located two xylosyl residues apart(Busse-Wicher et al. 2016). Despite the indication that AGX has a repeating pattern of monomers along its backbone, as our work is focused on the interaction between AGX and water and as water in these environments does not possess a long-range crystalline structure, the repeating pattern of monomers of AGX is not considered relevant. Therefore, for the current study, a random polymer consisting of 67% xylose, 20% glucuronoacid-xylose and 13% arabinoxylose is used.

The three types of monomers are constructed using Material Studio 8.0 and randomly polymerized into chains with a degree of polymerization of 100(Gorshkova et al. 2012). Gromacs 2016 software(Berendsen et al. 1995; Abraham et al. 2015) and Gromos 53a6 force field(Oostenbrink et al. 2004) are used for the MD simulation. Force field parameters of the monomers are obtained from an automated topology builder(Malde et al. 2011). Long range coulombic interaction is treated using the particle-mesh Ewald method. Five chains of AGX are inserted randomly into a periodic box, with periodic boundary conditions in the three directions to avoid finite size effects. The system is relaxed using a Nose-Hoover thermostat and a Parrinello-Rahman barostat. The temperature and pressure are set to 300 K and 0 Pa, respectively. The thermostat and barostat coupling time constants are both 0.5 ps. Relaxation is carried out for 20 ns with an integration time step of 1 fs. Both the total energy and system density converge within 1 ns and the system finally reaches a size of about 5$\times$5$\times$5 nm^3^ and a density of 1.3 g/cm^3^. The density is in accordance with the literature, as measured for a xylan powder extracted from corn cobs(Verbeek 2012). Following the same procedure, two more systems are prepared and then investigated to improve the statistics and obtain data more representative of disorder in real systems. The three repetition systems differ by the orientation and arrangement of chains but display all the same level of isotropy.

**Simulation of water sorption.** Gromos force field is designed to work with the single point charge (SPC) and SPC extended (SPC/E)(Berendsen et al. 1987) water models. We choose SPC because it reproduces better the saturation vapor pressure (4400 Pa at 300 K) than SPC/E does (1010 Pa at 300 K)(Errington and Panagiotopoulos 1998). Water adsorption is simulated at room temperature (300 K). Starting with the dry system, water molecules are inserted randomly one after another into the simulation box, avoiding overlap with the polymer and previously inserted water molecules. Each insertion is followed by an energy minimization and then a relaxation of 100 ps. Due to the computational costs, we choose to report results at 20 moisture content levels.

Methods of material properties measurement

**Uniaxial swelling strain.** In this study, we choose uniaxial instead of volumetric swelling strain, in accordance with most available experimental reports (as most samples are made in the form of thin films). The systems are equilibrated in NPT ensemble for 20 ns (*P*=0 Pa, *T*=300 K). All the thermodynamic variables, such as energy, temperature, pressure and volume, reach equilibrium in the first 1 ns. The uniaxial swelling strain is defined as

|  | $\epsilon_{X}\left( m \right)=\frac{x\left( m \right)-x\left( 0 \right)}{x\left( 0 \right)}$ | (1) |
| --- | --- | --- |

where $x\left( m \right)$ is the size of the system at moisture content $m$, and $x\left( 0 \right)$ is the size of the system in dry condition (i.e. Lagrangian approach). The uniaxial swelling strains of three orthogonal directions ($\epsilon_{X}$, where *X*=x,y,z) of the three repetition samples are measured and the average value ($\epsilon$) is reported.

**Determination of chemical potential and relative humidity.** The one-step perturbation (OSP) method is used to calculate the free energy (eq 2), which is needed to determine the chemical potential (eq 3) and, from there, relative humidity (eq 4). OSP was shown to successfully predict solvation free energies of a number of small polar and nonpolar solutes in water with sub-*k_B_T* accuracy and significantly greater efficiency than traditional stepwise calculation(Pitera and van Gunsteren 2001). It has also been used to successfully predict the adsorption isotherm of amorphous cellulose(Kulasinski et al. 2014).

The systems at different moisture contents are simulated in the isothermal-isobaric (NPT) ensemble for 20 ns (*P*=1 Pa, *T*=300 K). Then, the equilibrium of the system is perturbed by removing one water molecule while all the other molecules remain. The potential energy difference of the states before and after the perturbation is calculated. The time average of the free energy difference $\left. \left\langle\Delta F_{i} \right. \right\rangle_{t}$ is estimated according to the Zwanzig formula:

|  | $\left. \left\langle\Delta F_{i} \right. \right\rangle_{t}=-RTln\left\langle e^{-\frac{\Delta V_{i}\left( t \right)}{RT}} \right\rangle_{t}$ | (2) |
| --- | --- | --- |

where $R$ is the gas constant, $T$ is the temperature, and $\Delta V_{i}\left( t \right)$ is the potential energy difference induced by removing the $i$-th water molecule.

The chemical potential, $\mu,$ is the entity average of time-averaged free energy:

|  | $\mu\left( m \right)=\frac{\partial F}{\partial N}=\frac{1}{N\left( m \right)}\sum_{i=1}^{N\left( m \right)} \Delta F_{i}$ | (3) |
| --- | --- | --- |

where $N$ is the total number of water molecules in the system and $m$ is the moisture content. The relative humidity (RH) is related to the chemical potential as follows:

|  | $RH=e^{\frac{\mu-\mu^{*}}{\mathrm{RT}}}$ | (4) |
| --- | --- | --- |

where $\mu^{*}$ is the saturation chemical potential which is taken as the chemical potential at maximum moisture content(Gröndahl et al. 2003, 2004).

**Integral heat of adsorption**. Adsorption is an exothermic process and the enthalpy released by adsorption is an important description of the interaction between adsorbate and adsorbent. There exist several definitions for the heat of adsorption. Here we use the integral heat of adsorption of water vapor, which is the total heat released by water vapor being adsorbed, normalized by the total adsorption amount. Measurements are carried out in NPT ensemble (*P*=1 bar, *T*=300 K). In the heat of adsorption at a specific moisture content *m*, three contributions are involved, i.e. dry polymer (*p*), water vapor (*wv*) and the composite system (*c*) formed by water being adsorbed on the polymer. The integral heat of adsorption is defined as:

|  | $Q\left( m \right)={(H}_{p}+H_{wv}-H_{c})/n_{water}$ | (5) |
| --- | --- | --- |

where $H_{p}$, $H_{wv}$, $H_{c}$ and $n_{water}$ are the enthalpies of dry polymer, water vapor, composite and the amount of water in mole, respectively. Since the deformation of pure polymer induced by adsorption is generally neglected for simplicity, in this study, dry polymer is obtained by removing all water molecules in the composite system while keeping the configuration of the polymer. $H_{p}$ and $H_{c}$ are calculated with the definition of enthalpy $H=U+PV$, where *U*, *P* and *V* are directly measureable from MD. As the water vapor can be seen as an ideal gas with an acceptable error(Israel Urieli 2018), the $H_{wv}$ equals to 4$n_{water}$*RT*, because the internal energy of water vapor in ideal gas state approximated by the equipartition theorem is $\frac{6}{2}n_{water}RT$ (three translational and three rotational degrees of freedom), and the *PV* term estimated by equation of state of an ideal gas is $n_{water}RT$. Usually, heat of adsorption is reported as the heat released per kilogram of adsorbed water, therefore the unit of *Q* is converted from kJ/mol into kJ/kg by the factor of the molar mass of water (0.018 kg/mol). The integral heats of adsorption of the three repetition systems are collected.

**Uniaxial thermal expansion coefficient.** Thermal expansion simulation is carried out with a series of 2ns NPT (*P*=0 Pa, various *T*) simulations at 9 different temperatures, i.e. 296 K, 297 K, 298 K, 299 K, 300 K, 301 K, 302 K, 303 K and 304 K. All temperatures are in the vicinity of room temperature (300 K). The uniaxial thermal expansion coefficient as a function of moisture content $m$ is defined as:

|  | $\alpha_{X,T_{0}}(m)=\left. \frac{1}{x(T_{0},m)}\frac{dx(T,m)}{dT} \right\vert_{p}$ | (6) |
| --- | --- | --- |

where $x(T,m)$ is the length of the system along one of the three principle axis at temperature $T$ ( $T_{0}=300 K$). $\alpha_{X}$ is measured in three orthogonal directions for all three repetition systems and the average value is reported.

**Heat capacity.** Like for the determination of the thermal expansion coefficient, heat capacity is determined by a series of 2ns NPT (*P*=0 Pa, various *T*) simulations at 9 different temperatures, i.e. 296 K, 297 K, 298 K, 299 K, 300 K, 301 K, 302 K, 303 K and 304 K. The total energy $U(T,m)$ and volume $V(T,m)$ are recorded and the heat capacity of specific moisture content is defined as:

|  | $c_{p,T_{0}}(m)=\left. \frac{dU(T,m)}{dT} \right\vert_{p,T_{0}}+p\left. \frac{dV(T,m)}{dT} \right\vert_{p,T_{0}}$ | (7) |
| --- | --- | --- |

where $c_{p}$ is the heat capacity, $T_{0}=300 K$, *P*=0 Pa. The heat capacities of the three repetition systems are collected.

**Elastic constants and Poisson’s ratio.** The elastic constants are determined from the slope of the linear regime of stress-strain curves at room temperature. To construct the stress-strain curve for bulk (*K*), Young’s (*E*) and shear moduli (*G*), tensile volumetric, uniaxial and shear strains are applied respectively and the resulting stresses are collected. In the simulation results, the linear regime usually spans until a strain of 4%.

Although in principle the deformation rate does not play an important role as will be explained below, it is chosen as low as possible, namely 3.66 $\times$ 10^-5^ nm/ps, 5 $\times$ 10^-5^ nm/ps and 3.66 $\times$ 10^-5^ nm/ps, for bulk, Young’s and shear moduli measurements, respectively. The strain rate, therefore, is in the order of 10^8^ s^-1^. Stepwise strains are applied to a given structure with each step straining around 0.01%. After every step, a relaxation run of 100 ps follows, to allow molecular rearrangement. To mimic experimental mechanical tests, the relaxation conditions for bulk, Young’s and shear moduli are different. For bulk moduli, the strained structure is relaxed in the NVT ensemble. For Young’s and shear moduli, the structure is relaxed with the strained dimension fixed and the other dimensions coupled to the barostat (*P*=0 Pa). For example, in Young’s moduli measurement, if the sample undergoes uniaxial strain in *x* direction, then the following relaxation will be carried out with *x* fixed while *y* and *z* are coupled to a barostat to allow fluctuation. Due to the stepwise straining and the following relaxation, the influence of strain rate is minimized, which is advantageous. The disadvantage of this method is the much higher computational costs comparing with the static approach(Theodorou and Suter 1986) where the entropic contribution is neglected.

Besides direct measurement, the Poisson’s ratio and the shear moduli of isotropic materials, assuming homogeneity, can be predicted by bulk *K* and Young’s moduli *E* according to the equation:

|  | $\nu=\frac{3K-E}{6K}\mathrm{and}G=\frac{3KE}{9K-E}$ | (8) |
| --- | --- | --- |

The number of water molecules is constant during loading so that the measurements are conducted at constant moisture content, therefore the elastic constants measured correspond to the undrained ones. The moduli and Poisson’s ratios of the three repetition systems are collected. The Young’s and shear moduli of the three orthogonal straining directions are collected.

**Hydrogen bonds.** As HBs are playing a significant role in the hygromechanical behavior of the polymer[20, 21], we track their occurrence and distribution. The criteria for HB are defined by the configuration of the donor-hydrogen-acceptor triplet:

|  | $r\leq0.35 nm \mathrm{and} \alpha\leq30^{\circ}$ | (9) |
| --- | --- | --- |

where $r$ is the distance between the donor oxygen atom and the acceptor oxygen atom, and $\alpha$ is the angle of acceptor oxygen atom – donor oxygen atom – donor hydrogen atom. The interoxygen distance criterion of 0.35 nm refers to the first minimum of the radial distribution function of SPC water(Soper and Phillips 1986; Luzar and Chandler 1993). The angle of 30̊ is approximately the amplitude of vibrations that break HBs(Teixeira and Bellissent-Funel 1990). There are three types of HBs in hydrated systems, i.e. polymer-polymer, polymer-water and water-water.

Validation of the atomistic model

MD introduces several levels of simplification of the molecular interactions, such as harmonic bond, Lennard-Jones potential, etc. The validation of the numerical models, and especially comparisons with existing experimental data, is therefore necessary. Due to the fact that the properties of AGX suffer from a dearth of experimental reports, we choose density, isotropy, the adsorption isotherm and the swelling strains as our means of validation. It is already mentioned in the main paper that the density of the material corresponds well with experiments.

**Isotropy.** Wide-angle X-ray scattering (WAXS) experiments show that spruce xylan is isotropic under different humidity conditions(Escalante et al. 2012). In MD to characterize isotropy, the Hermans orientation function (HOF) which corresponds to nematic order parameter is used(Hermans and Platzek 1939). It is defined as:

|  | $HOF=\frac{3\left\langle{cos}^{2}\theta\right\rangle-1}{2}$ | (10) |
| --- | --- | --- |

where $\theta$ is the angle between the axis of interest and the direction of polymer monomer and the angular bracket denotes the entity average of all monomers in the system. Our simulation results show that HOF remains 0±0.015 regardless of moisture content, thus indicating the isotropy of the model at all moisture contents, which is in agreement with the WAXS results of(Escalante et al. 2012).

**Adsorption isotherm and swelling.** Using the methods described above for water sorption, the sorption isotherm and swelling strains of AGX are determined. Experimental sorption curves of different xylans, unfortunately not including AGX, are reported(Gröndahl et al. 2003, 2004; Kulasinski et al. 2016), but only(Kulasinski et al. 2016) reports swelling. Two adsorption branches of sorption isotherm of glucuronoxylan isolated from aspen wood are shown in Fig. S1a with white triangles(Gröndahl et al. 2003) and squares(Gröndahl et al. 2004). In terms of hygroscopic range and sigmoidal shape, our simulation results agree well with the experimental results. Many factors, such as exact chemical composition, the degree of substitution and the form of xylan sample (film or powder), can affect the measured sorption isotherms and may explain the slight differences observed in Fig. S1a. Experiments in dry state are known to have difficulty in removing the moisture totally, which might make the value of measured moisture content lower than reality. This could also explain the experimental isotherms having slightly lower moisture content under the same RH than MD.

The maximum moisture content in Fig. S1a is ~40%. In theory, when RH approaches 100%, the sorption isotherm becomes steep and the corresponding equilibrium moisture content tends to high values. In this study, the maximum moisture content being discussed is 70%.

Swelling is a frequent co-occurrence of moisture adsorption for biopolymers. Black dots in Fig. S1b are the uniaxial swelling strains, the arithmetical average of three repetition samples on three principal directions. The swelling strains are practically the same in all three principal directions which means that AGX swells isotropically. The grey dashed line is the linear fitting of black dots at low moisture content. The white triangles are the experimental swelling strain results for rye arabinoxylan(Kulasinski et al. 2016), which agree well with our simulation. Given that the sample is in the form of film, the experiment only covers *m*<15%. The swelling strains of a broader range of moisture content are solely measured through MD and shown in the inset of Fig. S1b.

a)

b)

Fig. S1 **a**) Sorption isotherms of xylan from MD simulation (black dots) compared with two experimental studies (triangles(Gröndahl et al. 2003) and squares(Gröndahl et al. 2004)). **b**) Uniaxial swelling strain versus moisture content $\epsilon_{X}\left( m \right)$ (black circles) compared with experimental results of rye arabinoxylan for *m*<0.15 (white triangles)(Kulasinski et al. 2016). The dashed line is a linear fit of the simulation data. The inset shows $\epsilon_{X}\left( m \right)$ for a broader range of moisture content, i.e. *m* = 0 ~ 0.7

Abraham MJ, Murtola T, Schulz R, et al (2015) Gromacs: High performance molecular simulations through multi-level parallelism from laptops to supercomputers. SoftwareX 1–2:19–25. doi: 10.1016/j.softx.2015.06.001

Berendsen HJC, Grigera JR, Straatsma TP (1987) The missing term in effective pair potentials. J Phys Chem 91:6269–6271. doi: 10.1021/j100308a038

Berendsen HJC, van der Spoel D, van Drunen R (1995) GROMACS: A message-passing parallel molecular dynamics implementation. Comput Phys Commun 91:43–56. doi: 10.1016/0010-4655(95)00042-E

Busse-Wicher M, Li A, Silveira RL, et al (2016) Evolution of xylan substitution patterns in gymnosperms and angiosperms: implications for xylan interaction with cellulose. Plant Physiol pp.00539.2016. doi: 10.1104/pp.16.00539

Den Haan R, Van Zyl WH (2003) Enhanced xylan degradation and utilisation by Pichia stipitis overproducing fungal xylanolytic enzymes. Enzyme Microb Technol 33:620–628. doi: 10.1016/S0141-0229(03)00183-2

Errington JR, Panagiotopoulos AZ (1998) A Fixed Point Charge Model for Water Optimized to the Vapor−Liquid Coexistence Properties. J Phys Chem B 102:7470–7475. doi: 10.1021/jp982068v

Escalante A, Gonçalves A, Bodin A, et al (2012) Flexible oxygen barrier films from spruce xylan. Carbohydr Polym 87:2381–2387. doi: 10.1016/j.carbpol.2011.11.003

Gorshkova T, Brutch N, Chabbert B, et al (2012) Plant Fiber Formation: State of the Art, Recent and Expected Progress, and Open Questions. CRC Crit Rev Plant Sci 31:201–228. doi: 10.1080/07352689.2011.616096

Gröndahl M, Eriksson L, Gatenholm P (2004) Material properties of plasticized hardwood xylans for potential application as oxygen barrier films. Biomacromolecules 5:1528–35. doi: 10.1021/bm049925n

Gröndahl M, Teleman A, Gatenholm P (2003) Effect of acetylation on the material properties of glucuronoxylan from aspen wood. Carbohydr Polym 52:359–366. doi: 10.1016/S0144-8617(03)00014-6

Hermans PH, Platzek P (1939) Beiträge zur Kenntnis des Deformationsmechanismus und der Feinstruktur der Hydratzellulose. Kolloid-Zeitschrift 88:68–72. doi: 10.1007/BF01518890

Israel Urieli (2018) Chapter 2b: Pure Substances: Ideal Gas (updated 1/17/11). https://www.ohio.edu/mechanical/thermo/Intro/Chapt.1_6/Chapter2b.html. Accessed 14 Dec 2018

Jacobs A, Larsson PT, Dahlman O (2001) Distribution of Uronic Acids in Xylans from Various Species of Soft- and Hardwood As Determined by MALDI Mass Spectrometry. Biomacromolecules 2:979–990. doi: 10.1021/bm010062x

Kulasinski K, Keten S, Churakov S V., et al (2014) Molecular Mechanism of Moisture-Induced Transition in Amorphous Cellulose. ACS Macro Lett 3:1037–1040. doi: 10.1021/mz500528m

Kulasinski K, Salmén L, Derome D, Carmeliet J (2016) Moisture adsorption of glucomannan and xylan hemicelluloses. Cellulose 23:1629–1637. doi: 10.1007/s10570-016-0944-8

Luonteri E, Siika-aho M, Tenkanen M, Viikari L (1995) Purification and characterization of three α-arabinosidases from Aspergillus terreus. J Biotechnol 38:279–291. doi: 10.1016/0168-1656(94)00139-4

Luzar A, Chandler D (1993) Structure and hydrogen bond dynamics of water–dimethyl sulfoxide mixtures by computer simulations. J Chem Phys 98:8160–8173. doi: 10.1063/1.464521

Malde AK, Zuo L, Breeze M, et al (2011) An Automated Force Field Topology Builder (ATB) and Repository: Version 1.0. J Chem Theory Comput 7:4026–4037. doi: 10.1021/ct200196m

Oostenbrink C, Villa A, Mark AE, Van Gunsteren WF (2004) A biomolecular force field based on the free enthalpy of hydration and solvation: The GROMOS force-field parameter sets 53A5 and 53A6. J Comput Chem 25:1656–1676. doi: 10.1002/jcc.20090

Pitera JW, van Gunsteren WF (2001) One-Step Perturbation Methods for Solvation Free Energies of Polar Solutes. J Phys Chem B 105:11264–11274. doi: 10.1021/jp012003j

Reid JSG (1997) Carbohydrate Metabolism: Structural Carbohydrates. In: Plant Biochemistry. Elsevier, pp 205–236

Soper AK, Phillips MG (1986) A new determination of the structure of water at 25°C. Chem Phys 107:47–60. doi: 10.1016/0301-0104(86)85058-3

Teixeira J, Bellissent-Funel MC (1990) Dynamics of water studied by neutron scattering. J Phys Condens Matter 2:. doi: 10.1088/0953-8984/2/S/011

Theodorou DN, Suter UW (1986) Atomistic Modeling of Mechanical Properties of Polymeric Glasses. Macromolecules 19:139–154. doi: 10.1021/ma00155a022

Verbeek C (ed) (2012) Products and Applications of Biopolymers. InTech

Yamasaki T, Enomoto A, Kato A, et al (2011) Structural unit of xylans from sugi (Cryptomeria japonica) and hinoki (Chamaecyparis obtusa). J Wood Sci 57:76–84. doi: 10.1007/s10086-010-1139-9
